# Cancer predictive studies

**DOI:** 10.1186/s13062-020-00274-3

**Published:** 2020-10-14

**Authors:** Ivano Amelio, Riccardo Bertolo, Pierluigi Bove, Eleonora Candi, Marcello Chiocchi, Chiara Cipriani, Nicola Di Daniele, Carlo Ganini, Hartmut Juhl, Alessandro Mauriello, Carla Marani, John Marshall, Manuela Montanaro, Giampiero Palmieri, Mauro Piacentini, Giuseppe Sica, Manfredi Tesauro, Valentina Rovella, Giuseppe Tisone, Yufang Shi, Ying Wang, Gerry Melino

**Affiliations:** 1 Torvergata Oncoscience Research Centre of Excellence, TOR, Department of Experimental Medicine, University of Rome Tor Vergata, via Montpellier 1, 00133 Rome, Italy; 2 San Carlo di Nancy Hospital, Rome, Italy; 3 Indivumed GmbH, Hamburg, Germany; 4 Medstar Georgetown University Hospital, Georgetown University, Washington DC, USA; 5 CAS Key Laboratory of Tissue Microenvironment and Tumor, Shanghai Institute of Nutrition and Health, Shanghai Institutes for Biological Sciences, University of Chinese Academy of Sciences, Chinese Academy of Sciences, 320 Yueyang Road, Shanghai, 200031 China; 6 The First Affiliated Hospital of Soochow University and State Key Laboratory of Radiation Medicine and Protection, Institutes for Translational Medicine, Soochow University, 199 Renai Road, Suzhou, 215123 Jiangsu China

**Keywords:** Neuroblastoma, Microbiota, Precision oncology, Omics

## Abstract

The identification of individual or clusters of predictive genetic alterations might help in defining the outcome of cancer treatment, allowing for the stratification of patients into distinct cohorts for selective therapeutic protocols. Neuroblastoma (NB) is the most common extracranial childhood tumour, clinically defined in five distinct stages (1–4 & 4S), where stages 3–4 define chemotherapy-resistant, highly aggressive disease phases. NB is a model for geneticists and molecular biologists to classify genetic abnormalities and identify causative disease genes. Despite highly intensive basic research, improvements on clinical outcome have been predominantly observed for less aggressive cancers, that is stages 1,2 and 4S. Therefore, stages 3–4 NB are still complicated at the therapeutic level and require more intense fundamental research. Using neuroblastoma as a model system, here we herein outline how cancer prediction studies can help at steering preclinical and clinical research toward the identification and exploitation of specific genetic landscape. This might result in maximising the therapeutic success and minimizing harmful effects in cancer patients.

## Background

Since the revelation of the whole human genome, there has been tremendous advances in sequencing technologies, with reductions of costs and time, allowing for an incredible step forward in the global cancer genomic fruition [1–3]. We moved from The Cancer Genome Atlas (TCGA) to the Pan-Cancer Analysis of Whole Genomes (PCAWG) Consortium [4–6], which includes most tumor types, matching DNA sequencing and RNA transcripts. Recently, 2658 cancers have been deeply analysed [7, 8] reconstructing the origin and evolution of mutational processes and driver mutation sequences of 38 types of cancer, including neuroblastoma. A significant number of driver gene mutations (4–5) was observed, and a fourfold diversification of these drivers and increased genomic instability have been reported at later stages.

Since the clinical application of massive sequencing, and moreover the clinical application of omics, each patient can provide an enormous amount of molecular data which can be also implemented in the drug discovery process, moving fast towards the selection of the right drug for the right patient. This attitude towards the introduction of precision medicine into clinical therapy, was furthermore boosted by President Obama’s speech introducing the Precision Medicine Initiative in 2015, aimed at creating a collaborative academical environment [9, 10] to improve the use of the omics in both patients’ treatments and drug development.

**Fig. 1:**
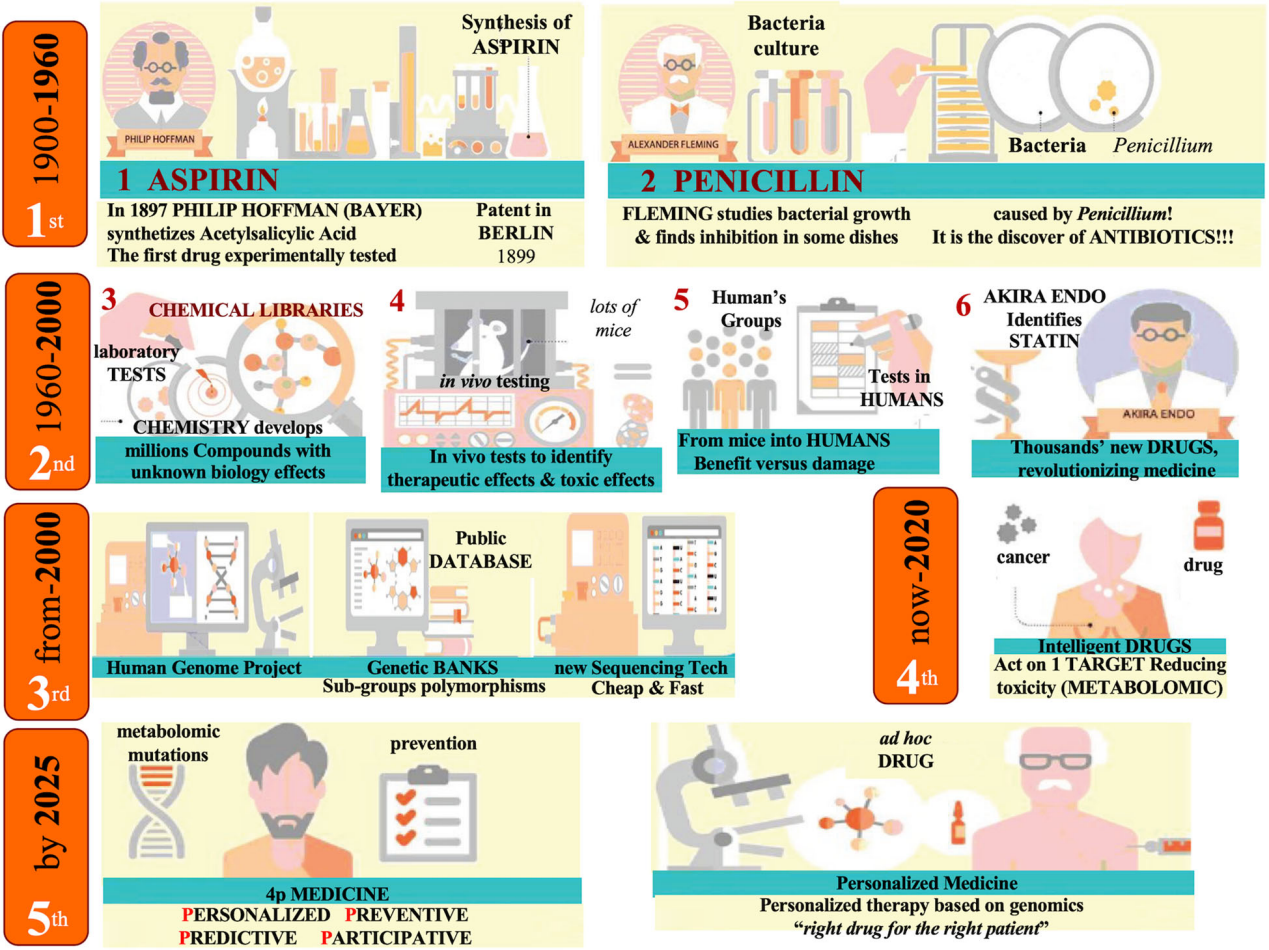
Evolution of Drug Development. We may distinguish 5 distinct phases in the evolution of drug discovery. In the first half of the last century, that was mainly occurring by serendipity (phase 1); nonetheless important drugs were identified, including Aspirin by Hoffman and penicillin by Fleming. In the second part of the last century (phase 2), the development of massive chemical libraries that could be tested in vivo in mice, subsequently translated into selective human groups, has allowed the definition of thousands new drugs that have revolutionized medicine, especially cancer therapy. In this century, we are equipped with the sequence of the entire human genome and large numbers of genetic banks, with specific mutations, deletions, polymorphisms and histone modifications (phase 3). This permitted the identification of intelligent drugs, acting only on one single target and therefore wanton toxicity (phase 4), in other wards, selecting the specific drug for the individual patient. With metabolic mutation, identification of predisposing mutations, selection of monitoring or predictive cluster of genes, proteins or phospho-protein, oncology will enter the 4P medicine: Preventive, Personalized, Predictive, Participative

Alongside with the advances in sequencing technologies, drug discovery rapidly reshaped itself. Drugs were initially discovered in a serendipitous fashion as in the case of penicillin [11], or aspirin [12]. Pharmaceutical efforts were aimed at finding molecules suitable for a given unmet medical need, as in the case of infections or inflammatory diseases. A single agent was tested in a single disease at a time. Chemical libraries speeded up the process, allowing the testing of multiple molecules in a relatively cheap and fast approach, and leading to the discovery of many classes of drugs which now have a predominant role in the clinics, such as statins [13]. At this point, the scientific focus was still on the molecule itself and the pathology for which it was developed. Patients’ clinical characteristics were still not fully evaluated during the drug development process, due to the yet inadequate availability of patient-derived molecular data. Nevertheless, further advances in the technologies used in this disease-oriented drug discovery approach, such as fragment screening of chemical compounds [14, 15] and lately cryo-electron microscopy [16] or structural bioinformatics [17], led to the development of a large number of therapies. This abundance of molecules, however, does not reflect the higher complexity of many diseases, among which cancer represents a prominent example: in many clinical contexts there is an abundance of therapies which can be chosen, but in many cases patients would not respond primarily [18]. Understanding the molecular profile of each of those primary refractory patients might represent a possible way to overcome our inability to tailor the right treatment for the right patient. This is in desperate need for primary refractory patients, as well as for patients who would benefit from many of the existing treatments but without knowing which one would get to the maximum clinical efficacy. Figure 1 reports a brief outline of the evolution of therapeutic drug development.

Massive sequencing approaches applied to disease entities such as cancer, have changed our point of view on diseases themselves, initially considered as entities per se, to disease as a single individual related clinical condition; this paradigm shift allowed to move to a completely patient-oriented effort to select the right drug, at the right moment. Effectively, under this light, many clinical trials have undergone a revolution: clinical efficacy is not anymore the only primary objective since also the study of biomarkers, to define subpopulations of treated or to be treated patients who would benefit more from a given treatment, is now performed [19, 20]. The vast availability of molecular data from patients, derived from multiple omics approaches, together with the implementation of computing algorithms and artificial intelligence, also opened a new era in drug discovery and clinical practice [21–23]: the selection of the right molecular target can be done on a single patient basis, and its efficacy and potential toxicity can also be predicted using a metabolomic approach in combination with genetic information [24].

Precision medicine has therefore entered the clinic in a single patient perspective [25–27]. This is an extremely actual approach, especially in disease entities which have been extensively characterized from a molecular point of view in the era of global sequencing approaches such as Neuroblastoma.

## The case of neuroblastoma

Neuroblastoma (NB) is clinically linked to distinct genomic abnormalities that seems to involve possible causative and progression genes of the disease [28–30]. These genomic abnormalities include deletions on chromosomes 1p, 11q or gains on 17q2,3; all being effective prognostic markers of the clinical outcome even though their molecular mechanisms are still not fully elucidated. Another crucial NB genetic signature is the amplification of the proto-oncogene MYCN; its amplification, with its over-expression, is a stronger predictor of tumor aggressiveness, chemotherapy refractoriness and clinical outcome. For example, NB patients with MYCN amplification report less than 50% 5 years survival, whilst the non-MYCN-amplified might report over 90%.

Nevertheless, MYCN is not the only culprit of NB aggressiveness. Recently, familial or sporadic NB patients have been reported to carry activating mutations of ALK. In the former cases, up to 50% of the cases show germline mutations in ALK gene, while sporadic NB may acquire ALK somatic mutations and ~ 2% display genomic amplification. ALK, member of the receptor tyrosine kinases superfamily and in particular, the insulin receptor (IR), shows homology with the leukocyte tyrosine kinase, the insulin-like growth factor-1 receptor kinase and the IR kinase. The single-chain transmembrane ALK is localized on human chromosome 2p23. At the molecular level, its mutation/amplification fosters cell proliferation and survival via the JAK–STAT, PI3K–AKT or RAS–MAPK pathways. Indeed, mutated (constitutively activated) ALK physically binds hyperphosphorylated ShcC, inhibiting, in response to growth factors, the MAPK signalling. Moreover, NB shows the deletion or loss-of-function mutation of the RNA-helicase ATRX. Interestingly, deregulation of ATRX and MYCN are mutually exclusive.

A further unexpected genetic rearrangement in high-risk NB is the activation of telomerase reverse transcriptase (TERT). This occurs in the chromosomal region 5p15.33 proximal of TERT. Again, TERT rearrangements, ATRX mutations and MYCN amplifications are mutually exclusive even though they take place exclusively within the high-risk NB patients. This leads to the concept that these genetic abnormalities converge on a similar function. Still, in MYCN-amplified tumors without TERT rearrangements, its expression is nevertheless increased: juxtapose TERT rearrangements to strong enhancers result in deep epigenetic remodelling of the regulatory region without changes in the gene copy number. Whole-genomic sequencing shows that ATRX mutations occur only in MYCN-non-amplified and TERT-normal NB and are associated with increase in alternative lengthening of telomeres (ALT). This indicates that telomere lengthening is a common trait of high-risk NB (MYCN-amplified, ATRX-mutated, TERT-rearranged cancers) independently from the underlying molecular mechanism of telomere maintenance. Therefore, high-risk NB show telomerase activation that is subsequent to either TERT rearrangement or MYCN amplification (which in any case is able to activates TERT).

The Chr17q region containing the TRIM37 gene is frequently amplified in neuroblastoma, as well as breast cancer. Since the acentrosomal spindle assembly following PLK4 inhibition, during mitotic division, depends on levels of the centrosomal ubiquitin ligase TRIM37 [31]. The steady state level of TRIM37 regulates the spindle assembly and subsequently the proliferation, in particular following PLK4 inhibition. Therefore, TRIM37 is a prognostic factor for human NB with 17p-deletion and, at the same time, an essential determinant of mitotic vulnerability to PLK4 inhibition [31]. This is highly relevant, as recently excellent PLK4 inhibitors have been identified [32, 33]. A PLK4 inhibitor, CFI-400945, triggers mitotic catastrophe in breast cancer cells overexpressing TRIM37 [34].

The locus deleted in 1p in NB contains an interesting gene, Trp73, codifying for the p73 protein [35, 36]. p53, p63 and p73 define the p53 family of transcription factors. All three are transcribed as several distinct protein isoforms [37–40]. Two alternative promoters drive the expression of either a transcriptionally active p73 (TA isoforms) proteins [41], containing a full N-terminal transactivation domain (TAD), or a N-terminally truncated (ΔN isoforms) proteins [42], that lack the TAD. ΔN isoforms might have fully independent functions or may act as dominant negative molecules by inhibiting the transactivating activity of the TA isoforms. For example, while TAp73 is an inducer of cell cycle arrest, neuronal function [43–45] and apoptotic cell death, and largely mimics the tumor suppressive activities of p53 [46–53], ΔNp73 isoforms promote cancer cell survival and exhibit oncogenic properties. The phenotypical characterization of selectively knockout mice for either TAp73 and ΔNp73 fully support their function as tumor suppressor or pro-oncogenic factors, respectively. Moreover, p73 is essential for the development and differentiation of the neuronal tissue. Accordingly, TAp73^−/−^ knockout mice as well as p73^D13/D13^ knockout mice show hippocampal dysgenesis with reduction of the neurogenesis in the subgranular zone of the dentate gyrus [54]. Conversely, ΔNp73^−/−^ knockout mice exhibit signs of neurodegeneration, as a consequence of the prosurvival function of this isoforms. Hence, TAp73 and ΔNp73 are crucial regulators of tumorigenesis and neurodevelopment [55, 56].

Recently, we discovered that expression of ZNF281, a zinc finger factor associated with several cellular functions, is deregulated in terminal differentiation of murine cortical neurons and in differentiating NB cells. Indeed, the mouse zinc finger transcription factor Zfp281 (or the human homologue ZNF281), involved in the control of neuronal progenitor stemness by inhibiting Nanog expression in mice through recruitment of the inhibitory complex NuRD on the Nanog promoter, is significantly expressed in neuronal cells, and significantly repressed during neuronal differentiation, including neuroblastoma [57]. ZNF281 is highly expressed in stage 4 NB patients supporting a role of ZNF281 in the progression of the disease. Accordingly, NB patients with “low-expressors”, thus indicating that ZNF281 represents a prognostic marker of human NB [57].

**Fig. 2:**
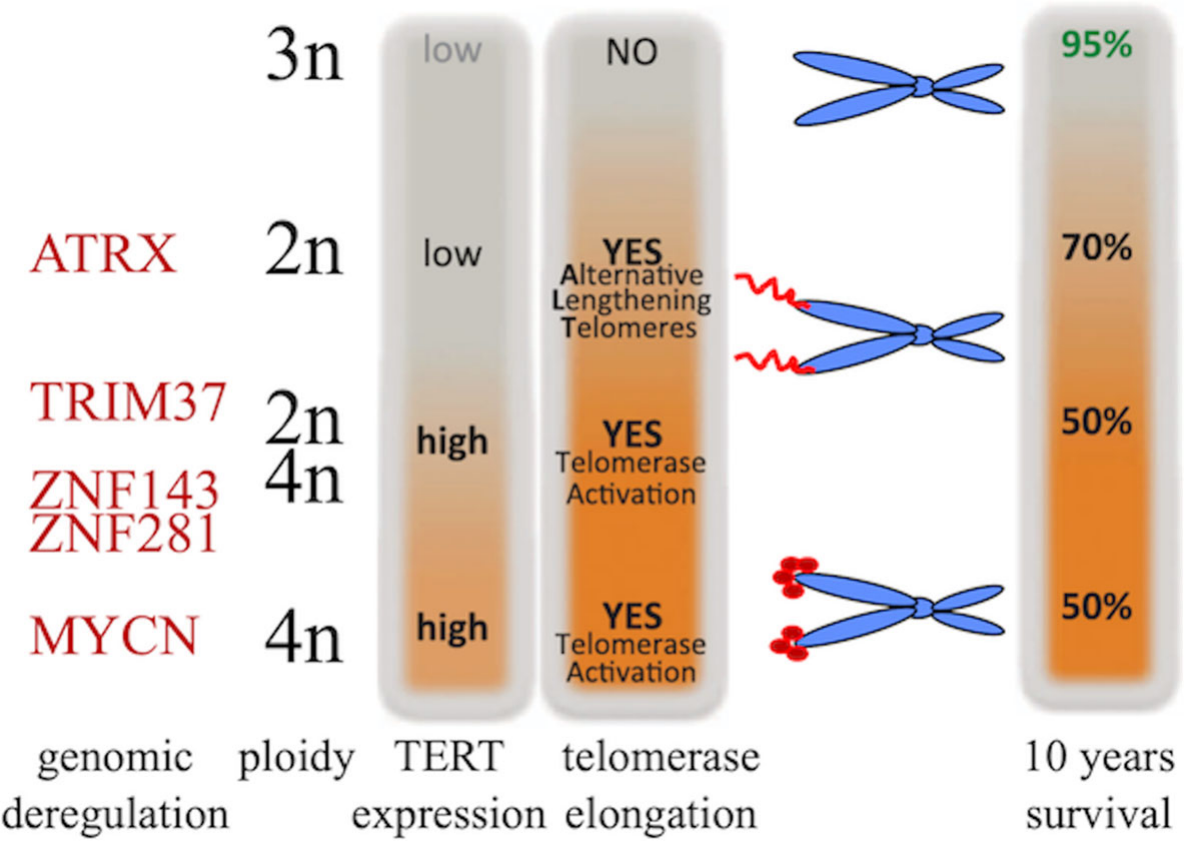
Risk groups for neuroblastoma patients. Depending on ploidy, TERT expression, telomerase elongation, ZNF281/ZNF143 expression, Chr17p or Chr1p deletions, neuroblastoma patients can be stratified into distinct sub-groups with distinct prognostic outcome. Therefore, the molecular identification of these markers is pivotal to define the most appropriate therapy for individual patient. For example, patients with Chr17p defect and impaired TRIM37 may be specifically selected for using PLK4 inhibitors, that would be otherwise ineffective in other patients

Another member of the same family is also expressed in NB tumours: ZNF143 [58]. In this case, LIN28B, or LIN28B mutant that is unable to inhibit let-7 processing, increases the penetrance of MYCN-induced neuroblastoma, potentiates the invasion and migration of transformed sympathetic neuroblasts, and drives distant metastases in vivo. In particular, LIN28B physically binds ZNF143 and activates the expression of downstream targets, that is GSK3B and L1CAM, affecting adhesion and migration of the cancer cells [58]. Figure 2 shows how the distinct NB subgroups with distinct genetic aberrations define individual prognostic groups.

It is therefore essential, following a diagnosis of NB, to understand which molecular defect is present in each individual patient, in order characterize the patient’s risk group and select the more appropriate therapy, accordingly. In each of these groups, the identification of the underlying molecular events at the bases of tumour progression [59–61] might allow for specific combination therapies to optimise therapeutic efficacy and minimise toxic side effects [62].

## Is there a role for exogenous factors such as microbiome?

The microbiome [63–66], often interacting with environmental urban life [67–70], constitutes a large mass of metabolizing bacteria, which are able to metabolise and transform normal constituents [71–73] and to impinge on the function of the host [74–77]. Just as examples, microbiota directly affects the B cell repertoire [78], the histone HDAC3 activity [79] or even the function of mutant p53 [80, 81]. Importantly, microbiota can also affect immunity to tumors and the efficacy of chemotherapies, but can also affect massively inflammatory cronic diseases [82, 83].

This, just to remain with the neuroblastoma example, are also of relevance in other cancer progression or even in neurophysiology. Accordingly, gut microbiota shows neuroprotective properties, reducing IL-6 secretion in different neural cell lines, including neuroblastoma [84, 85]. Here, these investigators identified two specific strains, *Parabacteroides distasonis* MRx0005 and *Megasphaera massiliensis* MRx0029, producing distinct C1-C3 or C4-C6 fatty acids [84] or, in another context, galacto-oligosaccharides [86] or short-chain fatty acid receptor 3 [87]. Similarly, *Roseovarius albus* increases brain derived neurotropic factor (BDNF) expression while reducing Bax/Bcl-2 ratio in neuroblastoma cell lines [88]. Neuroblastoma has per se the ability to impinge on the gut microbiota [89]. Consequently, it is conceivable to provide supplementary dietary treatment in neuroblastoma patients to improve the therapeutic response [90–93].

## Conclusion

The advance in technology, including massive sequencing, bioinformatic analysis by artificial intelligence, cloud computing, fast large scale proteomic and phospho-proteomic analysis is rapidly providing a unique opportunity to the global cancer genomic community to improve the previous analysis of TCGA with an individual systematic documentation of selective mutations which drive common tumour types. This provides, as being done now, the use of intelligent drugs with a single target for an individual patient, therefore reducing undesirable toxicity, while increasing the efficacy. In the near future, such possibilities will be expanded along with the use of the 4P medicine: Preventive, Personalized, Predictive, Participative.

## Data Availability

Not applicable.
